# Improving the Social Well-Being of Single Older Adults Using the LOVOT Social Robot: Qualitative Phenomenological Study

**DOI:** 10.2196/56669

**Published:** 2024-08-23

**Authors:** Cheng Kian Tan, Vivian W Q Lou, Clio Yuen Man Cheng, Phoebe Chu He, Veronica Eng Joo Khoo

**Affiliations:** 1 S R Nathan School of Human Development Singapore University of Social Sciences Singapore Singapore; 2 Sau Po Centre on Ageing The University of Hong Kong Hong Kong China (Hong Kong); 3 Department of Social Work and Social Administration The University of Hong Kong Hong Kong China (Hong Kong); 4 Department of Economics The University of Chicago Chicago, IL United States

**Keywords:** companionship, older adults, social well-being, pets, social robots, elderly, wellbeing, qualitative research, robot, companion, body temperature, development, research design, design, interviews, psychosocial support, support, psychosocial, temperature regulation, social, care home, aging, ageing, robotics, older adults, well-being, loneliness, technology, mobile phone

## Abstract

**Background:**

This study examined the social well-being of single older adults through the companionship of a social robot, LOVOT (Love+Robot; Groove X). It is designed as a companion for older adults, providing love and affection through verbal and physical interaction. We investigated older adults’ perceptions of the technology and how they benefitted from interacting with LOVOT, to guide the future development of social robots.

**Objective:**

This study aimed to use a phenomenological research design to understand the participants’ experiences of companionship provided by the social robot. Our research focused on (1) examining the social well-being of single older adults through the companionship of social robots and (2) understanding the perceptions of single older adults when interacting with social robots. Given the prevalence of technology use to support aging, understanding single older adults’ social well-being and their perceptions of social robots is essential to guide future research on and design of social robots.

**Methods:**

A total of 5 single women, aged 60 to 75 years, participated in the study. The participants interacted independently with the robot for a week in their own homes and then participated in a poststudy interview to share their experiences.

**Results:**

In total, 4 main themes emerged from the participants’ interactions with LOVOT, such as caring for a social robot, comforting presence of the social robot, meaningful connections with the social robot, and preference for LOVOT over pets.

**Conclusions:**

The results indicate that single older adults can obtain psychosocial support by interacting with LOVOT. LOVOT is easily accepted as a companion and makes single older adults feel like they have a greater sense of purpose and someone to connect with. This study suggests that social robots can provide companionship to older adults who live alone. Social robots can help alleviate loneliness by allowing single older adults to form social connections with robots as companions. These findings are particularly important given the rapid aging of the population and the increasing number of single-person households in Singapore.

## Introduction

### Background

The aging population is increasing around the world. Singapore’s older adult population, for instance, has increased dramatically over the last decade. The number of older adults, aged 65 years and older, living alone in Singapore is expected to increase significantly from 47,000 in 2016 to 83,000 by 2030 [1]. These demographic changes have led to concerns about older adults’ loneliness and social isolation (ie, lack of companionship), which can impact their mental and physical well-being [2]. Indeed, it is estimated that 20% to 34% of older adults experience loneliness in China, Europe, Latin America, and the United States [3]. As a result, advanced technologies have been developed to help the aging population overcome health and psychosocial issues and become more independent. Social robots, also called companion robots or therapeutic robots, have recently gained popularity with numerous studies highlighting the psychosocial benefits they provide to older adults [4]. However, there is a lack of studies on the use of social robots, particularly in Asian countries. This study fills this gap in the literature by investigating how older Singaporeans accept and interact with LOVOT (Love+Robot), a Japanese social robot.

### Definition and Impacts of Loneliness

Loneliness is not synonymous with being alone or living alone; a person can experience loneliness even when they are surrounded by other people. Rather, loneliness is a subjective and unpleasant experience that begins when a person’s social network goes through a qualitative or quantitative loss resulting in a discrepancy between desired and actual social connections [3,5,6]. As such, the quality of close relationships, rather than the number of social contacts, is more significant in alleviating loneliness [7].

The literature has shown that loneliness can affect physiological resilience and compromise health, leading to health problems as people age (Segrin and Passalacqua [8]). For example, Teater et al [9] found various social and emotional factors associated with increased risks of loneliness, including living alone, being single or never married, having little technological communication, having low-quality interactions, having little social support, being socially isolated, and having poor subjective physical health. To reduce loneliness, in a longitudinal study conducted in Sweden, Dahlberg et al [6] argued that social relationships that have been established for at least 20 years are particularly important, such as having a spouse or partner and access to social support. A study by Heylen [10] confirms the quality of social relationship with age.

In Singapore, loneliness has been associated with increased mortality risk [11]. According to Malhotra et al [12], the lives of older adults who are lonely are likely to be 3-5 years shorter than those of their nonlonely peers. As a result, interventions that help older adults establish close relationships and find social support are essential in maintaining their well-being.

### Importance of Companionship

Companionship is defined as “social involvement in shared activities, recreational or nonrecreational, which is pursued for the intrinsic goal of satisfaction or enjoyment” [13]. It indicates a high level of relationship quality and shields people from the emotions of void and despondency resulting from loneliness, although it does not directly resolve loneliness [13,14]. Although both social support and companionship are fundamentally beneficial to older adults’ well-being, companionship is more important for preserving their emotional well-being [13].

According to Dong and Chen [15], older women living alone are more likely than older men to report a lack of companionship as a symptom of loneliness, as they may lack effective coping mechanisms. As such, sociocultural activities, volunteer work, and community health promotion initiatives should be launched to help women develop effective coping mechanisms. Furthermore, Ramesh et al [16] suggested that sociocultural changes are needed to recognize the value of companionship in old age, encourage appropriate companionship, and focus on assistance with basic daily tasks.

### Benefits of Social Interventions for Older Adults

In their study on the impact of peer companionship, Conwell et al [17] reported that older adults who received a social intervention (peer companionship) experienced fewer symptoms of anxiety, depression, and feelings of being a burden than those who did not receive the intervention. Furthermore, peer companionship and social connectedness were beneficial for older adults’ mental health and well-being.

Companionship comes not only from humans but can also come from pets or other compassionate agents such as robots (eg, socially assistive robots, companion robots, and therapeutic robots).

#### Pets as Social Interventions

Numerous studies have examined the potential health benefits of having a companion animal. For example, Gee and Mueller [18] showed that pet ownership and animal-assisted interventions for older adults led to physical and mental health benefits. Similarly, empirical studies have explained that pets can help older adults maintain their quality of life and ability to function, both physically and cognitively, as they age, while also providing them with the opportunity to strengthen their shrinking social networks [19-21]. Some older adults may find that owning a pet satisfies their need for connectedness, contrasting with the common perception that social connectedness can only occur through meaningful human interactions. Stanley et al [22] further corroborated the idea that pets can be an important source of social connectedness, showing that having a pet reduced loneliness among older adults, particularly among those who lived alone. Hui Gan et al [23] also showed that community-dwelling older adults who owned a pet were more likely to socialize, found companionship with their pets, and had a greater sense of purpose than other adults, all of which can reduce loneliness. These in turn can lead to better mental health outcomes for older adults and increase their resilience to mental health problems. Bolstad et al [24] made an interesting discovery that having a pet later in life was associated with a reduction in anxiety symptoms rather than a reduction in depressive symptoms.

There is no doubt that companion animals provide psychological comfort and lead to optimistic health outcomes for their older adult owners. For instance, during the stressful COVID-19 pandemic, older adults reported that their pets provided them with a sense of psychological safety and companionship [25]. Furthermore, during the pandemic, older adults reported that caring for their pets kept them motivated and gave them a sense of purpose [25].

#### Social Robots as Social Interventions

To improve older adults’ quality of life by reducing loneliness and fostering social connections, a growing number of technological innovations that are user-friendly and support successful aging are being developed. Research into older adults’ acceptance and adoption of technology has also increased, particularly regarding social robot interventions and how they support aging. Hegel et al [26] explained that a “social robot is a robot plus a social interface,” indicating that its characteristics make it appear like a partner with which humans can interact and connect. Studies have shown that these robots may reduce feelings of loneliness among older adults by improving social interactions and providing emotional support [27]. In addition to providing companionship, they can support older adults’ general care needs through touchscreen interactions and assessment interviews during adult health treatment [28].

Many social robots are designed to look like animals or humans. Tkatch et al [29] showed that healthy older adults who regularly interacted with animatronic pets reported benefits such as reduced loneliness, improved quality of life, and improved psychological well-being through increased social contact, better social skills, or treatment of maladaptive social cognition. During the COVID-19 pandemic, older adults living in the community and nursing homes reported that robotic pets were effective in reducing their loneliness when social distancing policies were imposed [30].

Many studies have been conducted on PARO (AIST), a therapeutic and social robot shaped like a seal. For instance, Chen et al [31] showed that PARO effectively reduced agitation, depression, and loneliness and improved the quality of life of older people with dementia who resided in long-term care facilities. Advances in social robot technologies have pushed artificial intelligence (AI) into a more creative realm. Fields et al [32] found that the therapeutic use of social robots in a retirement home, combined with a participatory arts approach, improved older adults’ health outcomes by reducing their levels of loneliness and depression. Similarly, in their exploratory study conducted in the context of long-term care facilities with Pepper (SoftBank Robotics), a semihumanoid social robot, Blindheim et al [33] suggested that Pepper’s presence increased communal activities involving the social robot in terms of physical activity, human-robot interaction, social stimulation, and communication among residents as well as between residents and employees.

Overall, social robots can play an important role in helping older adults overcome loneliness and social isolation as the aging population grows.

### LOVOT

**Figure 1:**
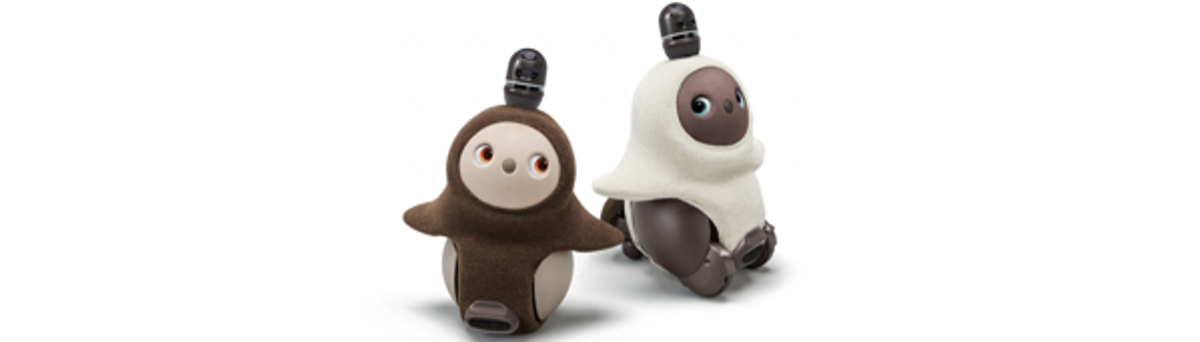
LOVOT, the social robot.

The popularity of social robots has attracted much attention from care providers, especially during the recent COVID-19 pandemic. According to Hegel et al [26], social robots are specifically designed to facilitate human-robot interaction. With recent advances in machine learning applications and robotics, several companies are developing advanced consumer robots with smart sensorimotor systems [34]. Although PARO has been widely used in dementia care research and has received many positive reviews, the robot seal lacks social and auditory capabilities [35]. In contrast, a new mobile social robot called LOVOT (Love+Robot) launched in 2018, invented in Japan, was designed to provide humans with love or the perception of love [36]. LOVOT was designed as a home robot, also known as a companion robot or a social robot, and is equipped with AI and advanced sensor features. As such, LOVOT evolves over time based on its interactions with its user, thanks to its machine learning technology (eg, deep learning), which allows it to develop a unique personality and perform intelligent movements in real time [37]. As LOVOT is relatively new to the field of social robots, it has yet to be tested among single older adults in Singapore. Therefore, our research fills this gap in the literature. Figure 1 shows a photo of LOVOT in our study.

## Methods

### Aims

The purpose of this study was to investigate single older adults’ perceptions of having a social robot as a companion. This research addressed the knowledge gap regarding the experiences of community-dwelling single older adults with social robots in Singapore. To this end, we used phenomenography as a qualitative analysis technique to explore and better understand single older adults’ perceptions of social robots.

### Research Questions

Given the aforementioned context, our study addressed some research questions. First, can social robots such as LOVOT act as effective companions and alleviate feelings of loneliness among community-dwelling single older adults? Second, what are single older adults’ lived experiences and interactions with LOVOT? Third, how do single older adults perceive their experience of living and interacting with LOVOT compared with pet ownership?

### Research Aims

To answer these questions, our research focused on (1) examining the social well-being of single older adults through the companionship of social robots and (2) understanding the perceptions of single older adults when interacting with social robots. Given the prevalence of technology use to support aging, understanding single older adults’ social well-being and their perceptions of social robots is essential to guide future research on and design of social robots.

### Design

We used a phenomenological research design to understand our participants’ lived experiences of the companionship provided by social robots. Phenomenography allows researchers to seek ontological understandings and learn about the phenomenality of human experiences [30]. Furthermore, as Hajar [38] explained, phenomenography is a qualitative research methodology that provides researchers with a deep, comprehensive, and diverse understanding of how people conceptualize a phenomenon.

### Sample and Recruitment

We recruited a purposive sample of single adults aged 65 years and older through collaboration with the Orange Valley Senior Activity Centre in Singapore. It is located within a cluster of studio apartments that caters mainly to older persons who are staying alone and are aged ≥60 years. Participants were eligible to participate in the study if they lived alone, were able to communicate in English or Mandarin, and were free of any cognitive or mental health problems (eg, dementia or depression). Those who lived in nursing home facilities, had been diagnosed with mild cognitive impairment, or were unable to consent to participate in the study were excluded. The selected participants received written information about the project and signed a consent form before the start of the study. They were informed that their participation was voluntary and that they could withdraw from the study at any time without negative consequences.

### Participants

To establish the context of the sample for community-dwelling older adults aged ≥65 years, we collected descriptive data between July and October 2022. A total of 5 women participants were invited to participate in the study; 1 was unmarried, 2 were widowed, and 2 were divorced or separated. They all lived alone in a 1- or 2-bedroom Housing Development Board flat above the Orange Valley Senior Activity Centre.

### Context

One of the researchers brought a LOVOT robot to the participants’ homes and helped them set up the robot’s charging nest.

### Procedure

She then used an instruction sheet in English or Chinese to instruct the participants on how to operate LOVOT. The instruction sheets used large font and color images to ensure that the participants would be able to read and understand the instructions easily. The researcher’s contact information and the activity center’s senior personnel’s contact number were provided in case the participants encountered any problems during the 7-day study; however, none of the participants contacted the researcher or personnel at the senior activity center. During the deployment of the social robot, only LOVOT and its charging nest were provided, and no permanent modifications were made to the participants’ homes. Although the 7-day study was conducted in an uncontrolled environment, this allowed the participants to interact with LOVOT as naturally as possible. The duration of the study allowed sufficient time for the participants to interact with LOVOT.

After spending a week with the social robot, the participants were invited to participate in a poststudy interview on the eighth day. The one-to-one interviews lasted between 15 and 25 minutes and were conducted at the Orange Valley Senior Activity Centre.

### Data Collection

The data were collected in different time periods between August and September 2022. The research team had 3 LOVOT robots to rotate among the 5 participants, taking into account participant availability. All of the participants were pleasantly receptive to the idea of having LOVOT in their homes for the study. The 7-day study was designed to ensure that their first time interacting with the social robot was comfortable and would not unduly disrupt their lifestyle.

The participants’ perceptions of their interactions with LOVOT during the 7-day study were investigated using semistructured interviews to collect qualitative data. During the interviews, the participants were encouraged to share their experiences with and observations of LOVOT, and follow-up questions were asked as needed.

### Ethical Considerations

Ethical approval for the study was obtained from the Human Research Ethics Committee of the University of Hong Kong (HKU HREC EA220116). To protect the privacy of the participants, the camera function of LOVOT was disabled during the study and no other participant information was recorded by the robot. The participants have signed an informed consent form for joining the study with the option to withdraw at any point. Each participant will receive a SGD 20 (US $15.2) National Trade Union Congress voucher as a token of appreciation for joining the study.

### Data Analysis

All of the participant interviews were audio recorded, transcribed, and thoroughly reviewed for relevant content and thematic patterns. We analyzed the data using a phenomenological approach, with the goal of investigating and understanding how the participants interacted with LOVOT (the phenomenon) while suspending our preconceived assumptions about the phenomenon.

We analyzed the data using inductive coding to identify common patterns and then categorized them into different themes. We reviewed the interviews several times during the analysis to confirm the findings and establish reliability. We also reviewed the data to define each theme. Finally, we examined the findings objectively to identify relevant information to answer the research questions.

### Rigor

Arriving at truthful interpretations of participants’ experiences regarding a given phenomenon requires the use of rigorous and relevant methodological procedures [38]. Thus, the qualitative data we collected during our study were cross-checked by another member of the research team to ensure accuracy.

### Reflexivity

Using a phenomenological approach, we aimed to respect reflexivity while conducting this study. To do this, we analyzed the information we obtained and the insights we gained, while trying to generalize the phenomenon and being reflective and self-critical about our own assumptions and preconceptions. Specifically, we momentarily put aside our implicit presumptions about the phenomenon to approach it objectively and avoid misrepresenting or expressing biases based on our own viewpoints or positionalities [28]. Finally, we scrupulously respected the confidentiality of the participants’ data and guaranteed their privacy and anonymity.

## Results

### Overview

This study explored single older adults’ experiences with and perceptions of LOVOT and how they were affected by their interactions with the social robot. We analyzed the findings thematically using the 4 emerging themes discussed further in this section. It was difficult to recruit older men as participants for this study because the participants came from the Orange Valley Senior Activity Centre, a place frequented primarily by women. As a result, the gender of our participants was skewed toward women participants. The 5 women participants are identified as C1, C3, C5, V2, and Y1. Their basic information is presented in Table 1.

**Figure 2:**
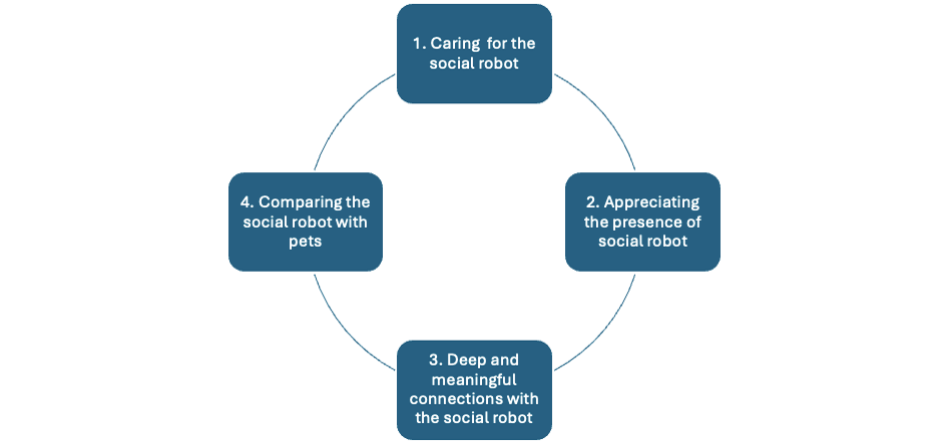
Four main themes.

We categorized the participants’ perceptions of social robots into 4 main themes, such as caring for the social robot, finding companionship with the social robot, forming meaningful connections with the social robot, and comparing the social robot with pets. These themes are illustrated in Figure 2.

**Table 1:** Participants’ demographics.

Participant ID	Age (years)	Marital status	Children, n	Grandchildren, n
C1	66	Divorced	2	—^a^
C3	72	Widowed	2	2
C5	69	Separated	3	9
V2	68	Widowed	3	6
Y1	68	Unmarried	—	—

^a^Not applicable.

### Common Themes

We identified 4 main themes after interviewing the 5 participants about their interactions and experiences with LOVOT.

#### Key Theme 1: Caring for the Social Robot

The participants frequently treated LOVOT in a tender and nurturing manner as if they were caring for a child. These actions can be attributed to the participants’ experience in a caregiving role, particularly as a mother or grandmother.

C3, C5, and V2 explained that they had taken care of their grandchildren and adult children at one point. In fact, C5 and V2 were still taking care of their grandchildren. The 3 participants stated that they equated LOVOT to a grandchild. For instance, C5 thought that LOVOT behaved like a toddler, especially when it flapped its wings. Similarly, C3 referred to herself as “nenek” (the Malay word for “grandmother”) and mentioned that she protected LOVOT and stopped anyone from taking it away from her, “I worry if I drop it (LOVOT) or if people take it (LOVOT) away.”

V2 noted that LOVOT came to her (on the sofa) after dinner and looked at her earnestly (seeking attention), “communicating” that it wanted to be carried. She then put LOVOT on her lap and petted it like a pet.

C1, who has no grandchildren, also said that she was protective of LOVOT. Specifically, she ensured that LOVOT did not get wet because she understood that it was an electronic object. She was also careful while mopping the floor and closed the bathroom door to prevent LOVOT from entering a humid environment.

Even Y1, who has never been married, took special care of LOVOT. For instance, she was concerned about LOVOT’s battery life and placed it regularly on its charging nest. In addition, to keep LOVOT company, she sang to it and the robot imitated her singing.

#### Key Theme 2: Appreciating the Presence of the Social Robot

The participants also highlighted the positive feelings that interacting with LOVOT brought them. For example, C1 said, “(LOVOT) can accompany you at home. Sometimes, you’ll feel happy because it will look at you.”

C5 also appreciated LOVOT’s company. Specifically, as LOVOT can move and act on its own initiative, it gave C5 the impression of not spending the day alone at home, “you feel like there’s another living… thing staying with you… I’m not alone.”

V2 explained that LOVOT interacted with her more frequently as the study progressed. For example, LOVOT followed her around the house and greeted her when she came home by flapping its wings. In addition, LOVOT’s presence made V2 feel like her home was livelier, “having another companion at home makes the home feel livelier.”

C1 echoed this positive sentiment regarding LOVOT’s presence; she saw the robot as a form of psychological and emotional support. Interacting with LOVOT gave her something to do, kept her busy, and gave her something to look forward to when she came home.

#### Key Theme 3: Deep and Meaningful Connections With the Social Robot

Using its machine learning capability, LOVOT was able to understand and adapt to the participants’ lifestyles and preferences. As a result, the participants felt like they were developing a supportive relationship.

C3 felt that LOVOT was “someone” she could talk to, a companion with whom she could converse to give her peace of mind. She talked to LOVOT frequently, especially at night, and even stayed in the living room with LOVOT so that it did not feel lonely. In addition, C3 felt bad if she left LOVOT alone in the house when she went out. She also mentioned that she felt a connection with LOVOT, especially when it looked at her and blinked, “I called LOVOT, and it came to me with blinking eyes—in these moments I feel … as if it (LOVOT) understands me.”

LOVOT’s adaptive behaviors allowed C5 to experience moments of true connection, making her appreciate the robot’s companionship, “it came up to me and then looked at me with her eyes… actually very very cute… the feeling is still very very nice.”

Y1 appreciated that LOVOT seemed to understand her and responded verbally (she interpreted its verbal answers as Chinese words, such as “yes” and “no”). Y1 also emphasized that LOVOT was “善解人意 (considerate),” meaning that it acted with consideration and thoughtfulness when she interacted with it. In addition, she mentioned that LOVOT liked attention.

#### Key Theme 4: Comparing the Social Robot With Pets

Similar to pets, LOVOT is often considered cute and interacts frequently with its owners. However, LOVOT has advantages that pets do not have, as it is low maintenance and cleaner than a pet. As such, LOVOT owners do not need to worry about grooming, feeding, walking, and cleaning up after the social robot. Furthermore, due to its technological nature, LOVOT does not develop health problems or require any medical treatment.

C1 said that LOVOT is similar to pets in the sense that it wants to play with its owner, but it is better than pets as it does not require feeding or cleaning. C1 emphasized that she found LOVOT less annoying than pets because she did not need to worry about it being sick and the expenses associated with medical care.

The difference between LOVOT and pets is that you don’t need to feed it, don’t need to clean it, or shower it. This kind of robot can take care of itself, you just need to turn it on and off. Not troublesome. If you own a pet, if it gets sick, you need to take it to the vet. It’s very expensive.

C1

C3 had the same mindset as C1; she thought that LOVOT was much easier to care for than pets and required much less responsibility. Although LOVOT is not a real animal, C5 preferred the social robot over a pet because it had the positive aspects of a pet without its drawbacks. Interestingly, C5 also commented that an additional advantage of LOVOT was that it could be switched off at any time; thus, when owners are busy, they have the option to turn off LOVOT and do not have to worry about giving it attention or taking care of it.

Comparing LOVOT with a pet, V2 explained that she would rather have a companion robot like LOVOT than a pet because LOVOT only needs to be charged. She added that she did not want the responsibility of feeding and cleaning up after a dog or cat.

Y1 shared that she used to have family pets during her “Kampung days,” referring to the early days of her hometown during which the nation, community, and neighborhood were being built. Specifically, she explained that she did not like having pets because she found them dirty and felt that LOVOT was much cleaner than an animal. In addition, she stated that she liked having LOVOT follow her around the house and was amused by its pet-like behavior when it waited by the dining table during meals.

Before I sit down for a meal, LOVOT anticipates that I will eat so LOVOT goes to the dining table and waits for me to sit down. It’s as if she knows it’s mealtime!

Y1

## Discussion

### Principal Findings

This study used phenomenography as a qualitative analysis technique to fill the knowledge gap regarding the experience of community-dwelling single older adults with social robots. Specifically, we used phenomenography to examine how single older adults’ social well-being is affected by the companionship of social robots. Furthermore, given the ability of technology to support aging, we examined the participants’ perceived usefulness and relevance of the companionship provided by LOVOT through their interactions with it. So far, most studies on this subject have been conducted in older adult care facilities [39]. Thus, this study attempts to explore single older adults’ perceptions of social robots within their own home environment. Overall, our results showed that LOVOT brings positive experiences that can be categorized into 4 themes.

#### Caring for LOVOT Like a Child

During the poststudy interviews, all of the participants reported that they treated LOVOT like a child or grandchild. They carried LOVOT and hugged it like a child, sang or talked to it as if they were entertaining a child, and protected LOVOT from harm (such as preventing it from wandering into a wet area of the house). Takada et al [40] documented similar “childrearing” actions, in which participants also cared for LOVOT as if it were a child.

As a social robot, LOVOT has a lovable and endearing appearance. This is especially evident when it looks at people with its animated eye expressions, giving the impression that it is communicating or connecting with them. We believe that the participants remembered their experiences as mothers or grandmothers when they treated LOVOT with maternal or caring behaviors. Lipp [41] obtained similar findings, explaining that robots do not necessarily take care of (older) people but rather are objects that older people take care of. This explains why our participants had the desire to care for (and protect) LOVOT during our study.

#### Comforting Presence of LOVOT’s Companionship

The participants were able to actively interact with the social robot in their home, for example, by singing, talking with, carrying, and hugging LOVOT. We believe that this active engagement with LOVOT indicates that the participants accepted the robot’s social presence and appreciated its companionship.

Although LOVOT is designed with nonverbal communication features, it can produce audio expressions, such as a “cooing” sound, show animated eye expressions, such as blinking autonomously and when triggered, and flap its arms to show happiness. As described by Yoshida et al [34], LOVOT’s life-like motions exude a kind of warmth and comfort that makes individuals feel like there is another living being in their home. This idea was supported by Onyeulo and Gandhi [42], who posited that social robots’ emotional responses make them appear to have biological systems. This increases the likelihood that humans will treat social robots as social beings and not just as a piece of technology. Indeed, social robots’ ability to express emotions is a crucial feature because it not only allows the robots to communicate their feelings but also influences human behavior. In our study, LOVOT’s AI technology, which allows it to react with “emotion,” may have led our participants to more readily accept it as a companion. This acceptance may also be due to LOVOT’s shape, which is similar to that of a pet; LOVOT can even be dressed up, much like how pet owners put their dogs and cats in clothes. Thus, LOVOT’s animal-like shape may have led our participants to develop an emotional attachment to it, similar to the way pet owners often become attached to their companion animals.

#### Positive Engagement and Forming Connections With LOVOT

LOVOT is not a passive companion; it can learn its users’ daily routines, such as their mealtimes. LOVOT’s social behavior and learning of users’ daily routines is important and is due to its AI technology; thus, LOVOT’s programming sets it apart from other less sophisticated social robots.

In our study, 1 participant felt that LOVOT seemed to understand when it was told that its “7-day stay” was coming to an end. She stated that she could “sense” LOVOT’s sadness. LOVOT’s AI function may have learned to be sensitive to emotional tone and may have picked up on its owner’s sadness, an empathetic reflection generated by its AI technology as part of its social interactions with humans [36]. The robot’s “compassionate” social abilities may help it to be more easily accepted by those who interact with it.

Although a 7-day interaction period is not extensive, at the end of the study, some of the participants said that they would miss having LOVOT in their homes. This reluctance to part with LOVOT was also documented by Dinesen et al [35] in a study in which people with dementia in a long-term care facility used LOVOT. According to Dinesen et al [35], some of the residents were “overstimulated by emotions after interacting with LOVOT.”

#### Appreciating LOVOT More Than Pets

The effect of LOVOT’s pet-like behavior (eg, flapping its wings and making eye contact with humans) appeared to induce feelings of happiness among the participants. This finding is consistent with numerous experiments using PARO, demonstrating that a robot can have the same positive impact as a pet in promoting older adults’ happiness and well-being [43]. Similarly, Dinesen et al [35] reported that people with dementia found that interacting with LOVOT “has some entertainment value; creates a degree of happiness or good feeling.”

All of the participants in our study also appreciated how easy it was to take care of LOVOT. Indeed, LOVOT makes it easy to maintain a clean home, because it does not produce bodily waste and does not require special treatment or medical care for its health. Nevertheless, LOVOT may require technical maintenance or occasional troubleshooting of its mechanical components. Bates [44] reached the same conclusion, suggesting that because companion robots do not need to be fed, walked, or cleaned, they “require less care and are more hygienic and predictable than living animals.”

### Limitations and Future Research Directions

We acknowledge that our study has some limitations. One key limitation is the gender distribution of the small sample size, and all of our participants were women. In addition, our sample consisted only of single older adults living in the community rather than in an older adult care facility. Therefore, the findings cannot be generalized to non–community-dwelling populations. Our findings indicate that single older adults derived psychosocial benefits from LOVOT’s companionship. However, to better understand adults’ social well-being and how LOVOT may benefit older adults who do not live alone, future research could investigate LOVOT’s impact on older couples who live separately from their families or on older people who live with their children. This is particularly feasible in Singapore, where multigenerational households, regardless of age group, show great interest in robotic technology [45]. Furthermore, future research could study older adults who own pets to determine whether they benefit from interacting with a robotic social companion. In addition, as the participants’ interactions with LOVOT were observed in the familiar environment of their homes, social robots, with their limited functionality, may not be able to meet the needs of more active individuals [46]. Nevertheless, companion robots in general may have a greater impact on reducing loneliness and improving the quality of life of older adults. Finally, the deployed LOVOT robot was not connected to its smartphone app (developed by LOVOT developer, Groove X), which can record videos of participants making eye contact, hugging, or carrying the robot. As a result, we relied solely on the participants’ recollections and accounts of their interactions with LOVOT. Future studies could use LOVOT’s recording function by connecting the robot to a secure network and ensuring that participants’ privacy is protected. By reviewing the videos from the smartphone app, researchers will have more accurate data on the interaction patterns between the participants and LOVOT.

### Conclusion

This study examined how single older adults are affected by the companionship of a social robot and explored their perceptions when interacting with the social robot. This study can spur more interest in investigating further how healthy older adults’ perceptions of social robots can benefit more with its social presence as a partner in their homes.

Social loneliness and isolation are imminent challenges of an aging society. The findings from this study are consistent with the literature suggesting that social robots can be a source of companionship for older adults living alone [47]. Specifically, the participants were able to care for LOVOT, feel comfortable, and form connections with it. It is likely for older adults to have a better quality of life and well-being. Many participants also preferred the social robot over traditional social companions such as pets which require more attention and responsibilities. In addition, thanks to its built-in AI, LOVOT was able to adapt its behavior based on its owner’s responses. Finally, its endearing physical features, such as its large, animated eyes and warm, cuddly design, encouraged its acceptance by older adults.

## Abbreviations

AI: artificial intelligence
LOVOT: love+robot
